# On the relation between oral contraceptive use and self-control

**DOI:** 10.3389/fendo.2024.1335384

**Published:** 2024-04-02

**Authors:** Alyssa C. Smith, Daniel Smilek

**Affiliations:** Department of Psychology University of Waterloo, Waterloo, ON, Canada

**Keywords:** oral contraceptives, self-control, self-regulation, assessment, locomotion

## Abstract

In two studies we examined the relation between oral contraceptive (OC) use and self-reported levels of self-control in undergraduate women using OCs (Study 1: OC group N = 399, Study 2: OC group N = 288) and naturally cycling women not using any form of hormonal contraceptives (Study 1: Non-OC group N = 964, Study 2: Non-OC group N = 997). We assessed the self-overriding aspect of self-control using the Brief Self-Control Scale (BSCS) and strategies for self-regulation using the Regulatory Mode Scale (RMS), which separately measures the tendency to assess one’s progress towards a goal (assessment), and the tendency to engage in activities that move one towards an end goal (locomotion). In Study 1, we found no significant differences between OC and non-OC groups in their levels of self-overriding or self-regulatory assessment. However, we found that those in the OC group reported significantly greater levels of self-regulatory locomotion compared to those in the non-OC group, even after controlling for depression symptoms and the semester of data collection. The findings from Study 2 replicated the findings from Study 1 in a different sample of participants, with the exception that OC use was also related to higher levels of assessment in Study 2. These results indicate that OC use is related to increases in self-regulatory actions in service of goal pursuit and perhaps the tendency to evaluate progress towards goals.

## Introduction

There is growing interest in understanding how various cognitive processes and tendencies might be influenced by commonly used oral contraceptives (see 1). The use of combination oral contraceptives (OCs)—which suppress endogenous levels of progesterone and estrogen via oral administration of synthetic versions of these hormones—has been associated with memory [e.g., (2–5)] and attention [e.g., (6, 7)] performance. It has been postulated that these relations occur because 1) various brain areas involved in cognitive processing contain sex hormone receptors and are responsive to changing levels of these hormones (8–11), and/or because 2) hormonal contraceptives alter the cortisol-based stress response, which has a knock-on influence on brain regions that respond to cortisol (12). Here we build on this prior literature by exploring the relation between OC use and self-control, which is broadly construed as the ability to “override” one’s own momentary impulses [e.g., (13, 14)] in the service of pursuing one’s valued goals (15). Our interest in the relation between OCs and self-control also stemmed partly from the common use of OCs (which are the most frequently used hormonal contraceptive; 16), and partly from the large body of prior work showing that self-control is linked to success in a variety of domains of everyday life, such as maintaining positive relationships, achieving physical and mental health, performing well in academics, and building personal wealth [see (13, 17, 18)].

To date, few studies have directly investigated the relation between OC use and self-control. Perhaps the most relevant study was reported by Zethraeus and colleagues (12). This study aimed to determine the influence of OC use on general well-being, and in so doing, included a putative measure of self-control as part of the Psychological General Well-Being Index [PGWBI; (19)]. In the study, women (aged 18-30 with regular menstrual cycles) willing to start using OCs were randomly assigned to three months of treatment with either an OC or a placebo. Participants completed the PGWBI prior to beginning treatment (baseline) and again after the 3-month treatment period. Results showed that scores on the measure of self-control (in the PGWBI) after treatment were significantly lower than baseline in the OC group, but not in the placebo (non-OC) group, suggesting that OC use reduces self-control. While these results are intriguing, there are several key limitations to this study as it pertains to the relation between OC use and self-control. First, the items of the PGWBI assessing self-control do not uniquely index self-control, but seem to assess aspects of mental *stability* as well^1^. Second, there was no direct statistical comparison of the pre- to post-treatment change scores between the OC and placebo groups, nor was there a statistical comparison of the levels of self-control following treatment across groups. And third, there was no attempt to control for symptoms of depression, which are known to vary with OC use (20). Unfortunately, these limitations preclude any strong conclusions about the relation between OC use and self-control from the study reported by Zethraeus et al. (12).

Other work has examined the relation between OC use and various constructs that are related to self-control [e.g., (5–7)]. For example, Bradshaw et al. (7) examined the relation between hormonal contraceptive (HC) use (including OCs) and *perseverance* during self-paced cognitive tasks, which is relevant because presumably, perseverance involves some degree of self-control [e.g., (21, 22)]. In one of their studies, participants were presented with two similar images side-by-side and asked to “find the differences between the left and right images” [(7); p. 3]. They were then told they could move to the next page whenever they wanted. The time participants spent searching for differences between the images was operationalized as perseverance, while the number of differences correctly identified was taken as an index performance. For present purposes, the key finding was that compared to the non-HC users, HC users persevered *less* (i.e., spent less time on the task), and as a result, these individuals performed significantly worse (detected fewer differences between images) [cf. (23)]. While there might be many reasons why HC users persevered less than non-HC users, one explanation is that HC users have poorer self-control than non-users.

Evidence suggesting a possible relation between OC use and self-control also comes from neuroimaging studies. Brain imaging and stimulation studies have implicated the prefrontal cortex (PFC) in the execution of self-control (24–26), and there is evidence of different functional connectivity within this region when self-control is engaged successfully versus when it fails (27). Interestingly, the PFC also has a dense population of sex hormone (e.g., estrogen and progesterone) receptors (8–11), suggesting that the PFC – and perhaps its related functions (such as self-control) – may be sensitive to changes in these hormone levels. There is also evidence that use of hormonal contraceptives is associated with differences in functional connectivity involving the PFC (28, 29).

## The present studies

Building on previous research, in the present studies we sought to directly explore the relation between OC use and self-control. We focused specifically on OC use rather than the broader category of hormonal contraceptive use because 1) as we noted, OCs are the most commonly used hormonal contraceptive (16), and 2) oral delivery of exogenous hormones could be associated with different metabolic and cognitive effects than other delivery methods (30). Importantly, as the present investigation is one of the first to directly examine the relation between OC use and self-control, we aimed to simply determine whether OC use and self-control are correlated with one another, leaving issues of causality to be addressed by future studies.

Our exploration involved a secondary data analysis of a large data set collected as part of a beginning-of-term survey completed by undergraduate students at the University of Waterloo. Included in this survey were measures of OC use as well as two useful measures of self-control, which captured quite different aspects of the broad construct of self-control. One of the measures was the Brief Self-Control Scale (13), which indexes a person’s ability to inhibit or override his or her automatic impulses (i.e., *self-overriding*). The other measure was the Regulatory Mode Scale (31), which assesses a person’s tendency to self-regulate when pursuing his or her goals. Specifically, the Regulatory Mode Scale evaluates two dimensions of self-regulation: 1) *assessment*, which refers to a person’s tendency to evaluate, prioritize, and assess progress of competing goals; and 2) *locomotion*, which refers to a person’s tendency to initiate and maintain goal pursuit. While there are limitations to using self-report measures, there is evidence that self-reports of self-control are better predictors of self-control related behaviors (e.g., physical activity) than some behavioral measures (32).

Fortunately, the data set also included a measure of depression [as part of the Depression Anxiety Stress Scale-21; (33)], which allowed us to examine the relation between OC use and self-control while controlling for symptoms of depression. Controlling for depression in this context is important given previously established relations between mood and self-control [e.g., (34, 35)], as well as between mood (particularly depression) and OC use (20, 36–40). It turns out that the latter association has been debated in the literature: Some studies report that OC use is related to *increased* positive mood (37, 38, 40); other studies report a detrimental effect of OC use on affect (20); and still others showed no difference in negative affect across OC users and non-users (36, 39). In any case, given these possible associations, we thought it would be prudent to control for depressive symptoms when assessing the link between OC use and the self-control measures included in our samples.

Finally, as the measures relevant to our study were included in multiple semesters of data collection, it was possible to aggregate the data to achieve reasonably large samples and also to test for replicability. Accordingly, Study 1 focused on analyses of data collected during the Winter and Spring semesters of 2020, which then served as the foundation for a pre-registered replication. Study 2 was the replication and focused on analyses of data from Fall 2020 and Winter 2021. Because the data were collected across semesters as the COVID-19 pandemic was evolving, we statistically controlled for semester of data collection when assessing the links between self-control and OC use in each study.

## Study 1

## Method

### Participants

Participants were undergraduate students at the University of Waterloo who indicated their sex as female. Participants received partial course credit in exchange for completing the Pre-screen and Mass Testing surveys (see below). Participants were excluded from each semester based on the criteria outlined in the Data Cleaning section. The cohort in Winter 2020 initially consisted of 1576 females, of which 509 participants were excluded leaving 1067 participants, with 315 using OCs and 752 naturally cycling. The Spring 2020 sample included 642 females. Three hundred and forty-six (346) were excluded, leaving 296 participants, of which 84 participants were using OCs and 212 were naturally cycling. Our final sample for Study 1 included a total of 1363 participants, with 399 using OCs and 964 naturally cycling and not using any form of hormonal contraceptives. These participants had a mean age of 20 years old. While all participants reported their sex as female, gender identities included gender queer/gender non-conforming/gender non-binary (N = 8), non-binary woman (N = 1), two-spirited (N = 2), man/transman (N = 2), and woman/transwoman (N = 1340). Ten participants did not report a gender identity.

### Pre-screen and mass testing surveys

Data for this study were collected as a part of a Pre-screen survey and the Mass Testing survey at the University of Waterloo. The Pre-screen and Mass Testing surveys include a series of questions administered online in close succession at the beginning of each semester using Qualtrics software. Undergraduates enrolled in psychology courses at the university were eligible to complete this survey. As part of the Pre-screen survey participants were asked to provide their biological sex, age, and to answer the following question about hormonal birth control use: “Are you currently using one of the following methods of birth control?” Participants responded by selecting from a list that included: oral contraception (i.e., birth control or “the pill”), birth control patch, vaginal ring, birth control injection, IUD^2^, hormonal implant, none of the above (in Winter 2020 this option was ‘does not apply to me’), and prefer not to answer. Participants were also asked to specify whether they were currently being treated for depression or anxiety, whether they were currently using medications for psychosis, and the date of their last menstruation. The Mass Testing survey involved the administration of a large battery of questionnaires, among which were the Brief Self-Control Scale, the Regulatory Mode Scale, and the Depression Anxiety and Stress Scale-21 (see descriptions below). Data was collected online at the University of Waterloo at the beginning of two semesters: Winter 2020 (January-February) and Spring 2020 (May-June).

### Data cleaning

For the present study, we utilized a similar data cleaning procedure as in our prior work [see (41), under review]. The R code for our analyses can be viewed at https://osf.io/84pc6/. The data cleaning procedure and exclusion criteria were determined *a priori* and were as follows: First, participants who were included in multiple semesters of data were only included in the analyses for the first semester in which they met the inclusion criteria. In subsequent semesters they were excluded from the analyses (to ensure each semester was an independent sample). Since Winter 2020 was the first semester of data cleaned, all participants were retained for this criterion. In Spring 2020, we excluded 138 participants who were included in the Winter 2020 sample. Participants were also excluded if they did not respond to the item inquiring about hormonal contraceptive use (N = 195; Winter 2020: N = 138; Spring 2020: N = 57), if they indicated they used a hormonal contraceptive other than the oral contraceptive pill (e.g., an injection, patch; N = 30; Winter 2020: N = 20; Spring 2020: N = 10), or if they indicated use of an IUD (N = 127; Winter 2020: N = 88; Spring 2020: N = 39). We then addressed minor variations in the wording of response options for the questions included in the present study. For instance, in one sample a response to a question about the use of birth control was “does not apply to me”, while the corresponding alternative the other samples was “none of the above”. These were treated as the same response. To avoid the potential confound of mental health, we then excluded participants who reported currently receiving treatment for depression, anxiety, or using anti-psychotic medications. One hundred and ninety-three participants were excluded for currently receiving treatment for depression (Winter 2020: N = 139; OC N = 76, non-OC N = 63; Spring 2020: N = 54; OC N = 25, non-OC N = 29), or declining to disclose whether or not they were currently receiving treatment for depression (Winter 2020: N = 12; Spring 2020: N = 5). Seventy-eight were excluded because they were currently receiving treatment for anxiety (Winter 2020: N = 55; OC N = 25, non-OC N = 30; Spring 2020: N = 23; OC N = 11, non-OC N = 12) and 9 were excluded for not disclosing whether or not they were currently receiving treatment for anxiety (Winter 2020: N = 7; Spring 2020: N = 2). Two participants (Winter 2020: N = 1; Spring 2020: N = 1) using anti-psychotics were also excluded. Next, we checked for and eliminated poor quality data based on participants’ patterns of responses. Participants were excluded from the analyses if 1) the number of clicks they made was fewer than the number of required responses per page of a survey (N = 1; Winter 2020: N = 0; Spring 2020: N = 1), 2) their responses were faster than 1-second per scale item (N = 35; Winter 2020: N = 25; Spring 2020: N = 10), and if 3) participants responded to fewer than half of the items on a scale (N = 9; Winter 2020: N = 9; Spring 2020: N = 0; note: responses on items for a given scale were averaged to arrive at the scale scores). Post-hoc, we excluded participants who did not report their birth year (N = 13) or were older than 45 years of age (N = 8) in order to ensure our sample consisted of females of reproductive age. This did not change the results in a meaningful way.

## Materials

### Brief self-control scale

The Brief Self-Control Scale (BSCS) is a 13-item measure used to assess self-control (13). Participants respond to statements such as “I am good at resisting temptation” and “I often act without thinking through all the alternatives” and rate them on how typical the statements are of themselves on a 5-point scale that included anchors such as 1 – *not at all* to 5 – *very much*. Eight items are reverse coded. Higher scores indicate more self-control.

### Regulatory mode scale

The Regulatory Mode Scale (RMS) is a trait-level measure of self-regulation strategies. It includes twenty-four items that are scored on a scale from 1 – strongly disagree to 6 – strongly agree. The RMS is scored on two sub-scales: locomotion and assessment. The locomotion subscale is scored from twelve items such as “I don’t mind doing things even if they involve extra effort”. Two of the twelve items are reverse coded. Higher scores indicate greater locomotion. The assessment subscale is scored from twelve items such as “I often critique work done by myself or others”. Three of the twelve items are reverse coded. Higher scores indicate greater assessment.

### Depression anxiety stress scale-21

The Depression, Anxiety, and Stress Scale (DASS) is a 21-item measure assessing symptoms of depression, anxiety, and stress over the previous week (33). Participants rate items such as “I felt that I had nothing to look forward to” (depression), “I was worried about situations in which I might panic and make a fool of myself” (anxiety), and “I found it hard to wind down” (stress) on a scale from 0 (*did not apply to me at all*) to 3 (*applied to me very much or most of the time*). The scale contains seven items related to each of depression, anxiety, and stress and higher scores on the scale items indicate higher levels of these experiences.

## Results and discussion

All analyses were performed in R (42). We used the psych, car, apaTables, and basic R packages to perform the Null Hypothesis Significance Tests. Anonymized data and analysis scripts will be available at https://osf.io/84pc6/. To determine whether there were differences between OC users and non-users we first conducted a series of planned comparisons using t-tests comparing those using OCs and those not using OCs on each of our measures of self-control^3^. To account for symptoms of depression and the semester of data collection, we conducted a series of hierarchical regressions predicting each of the measures of self-control with OC use while statistically controlling for symptoms of depression and semester of data collection.

### Descriptive statistics

**Figure 1:**
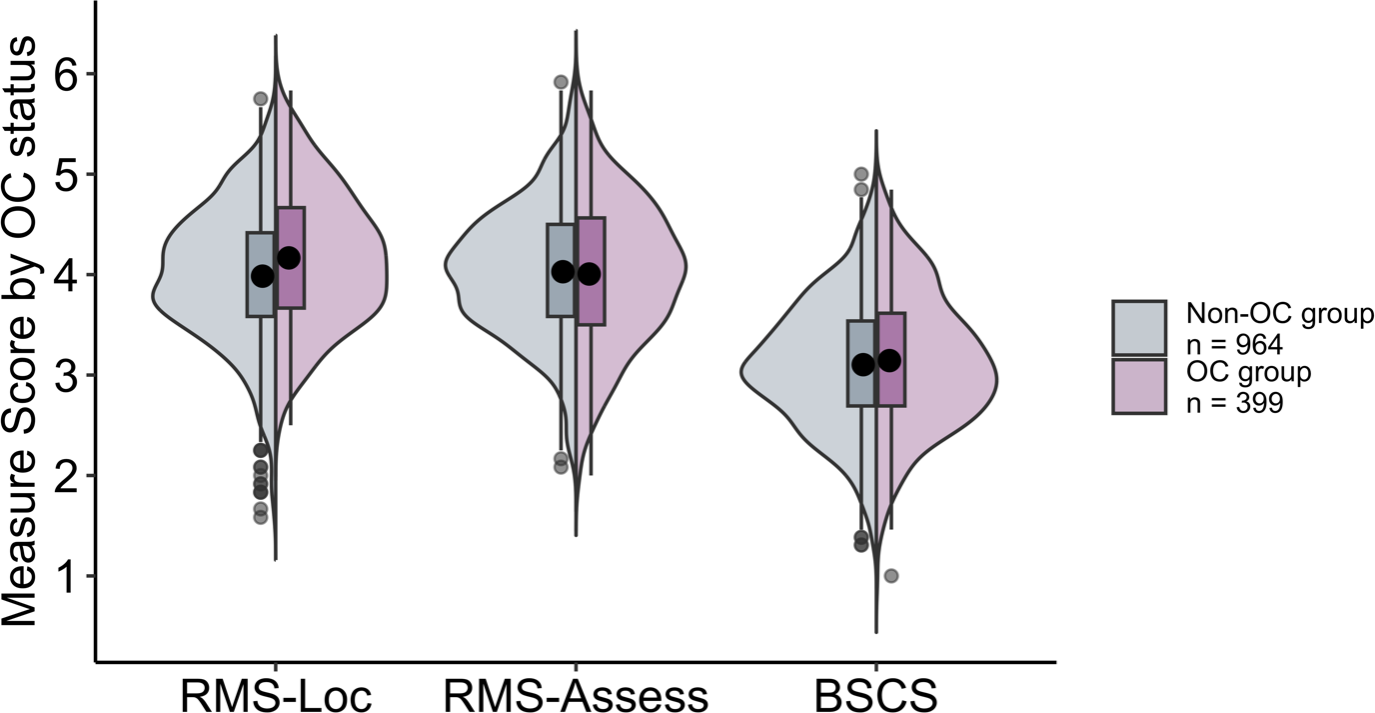
Split violin plots with box and whisker plots (boxplots) for each of the measures (RMS-Locomotion, RMS-Assessment, and BSCS) as a function of OC use in Study 1.

**Table 1** includes the descriptive statistics for each measure as a function of semester (Winter 2020 vs. Spring 2020) and group (OC vs. Non-OC). All scales showed high reliabilities, with Cronbach alphas of .76 or greater. Cronbach alpha values and Pearson correlations between the measures within each group are provided in **Appendix A** of the **Supplementary Materials** (**Tables A1, A2**). Boxplots depicting each of the attention measures as a function of OC use condition (averaged across semesters) are shown in **Figure 1**.

**Table 1:** Descriptive statistics of measures by semester and group for Study 1.

Semester	Group	Measure	N	Mean	SD	Skew	Kurtosis
Winter 2020	Non-OC group	BSCS	752	3.11	0.65	0.08	-0.13
		RMS-Loc	752	3.98	0.68	-0.33	0.39
		RMS-Assess	752	4.01	0.66	-0.08	0.06
		DASS-Dep	752	0.80	0.74	0.94	0.04
		DASS-Anx	752	0.72	0.66	0.97	0.23
		DASS-Stress	752	0.90	0.66	0.59	-0.34
	OC group	BSCS	315	3.11	0.69	0.07	-0.23
		RMS-Loc	315	4.13	0.67	0.03	−0.62
		RMS-Assess	315	3.98	0.71	-0.08	-0.36
		DASS-Dep	315	0.73	0.73	1.20	0.74
		DASS-Anx	315	0.68	0.64	1.15	0.94
		DASS-Stress	315	0.94	0.69	0.79	0.11
Spring 2020	Non-OC group	BSCS	212	3.06	0.63	-0.14	-0.46
		RMS-Loc	212	4.00	0.74	−0.21	−0.48
		RMS-Assess	212	4.09	0.59	0.19	0.10
		DASS-Dep	212	0.81	0.66	0.87	-0.11
		DASS-Anx	212	0.60	0.50	1.03	0.61
		DASS-Stress	212	0.87	0.58	0.64	0.04
	OC group	BSCS	84	3.29	0.68	0.13	-0.65
		RMS-Loc	84	4.31	0.65	0.39	-0.64
		RMS-Assess	84	4.09	0.81	-0.32	-0.37
		DASS-Dep	84	0.69	0.68	1.32	1.22
		DASS-Anx	84	0.55	0.51	0.95	0.30
		DASS-Stress	84	0.87	0.60	1.14	1.42

BSCS, Brief Self-Control Scale; RMS-Loc, Regulatory Mode Scale – Locomotion Subscale; RMS-Assess, Regulatory Mode Scale – Assessment Subscale; DASS-Dep, DASS Depression Subscale; DASS-Anx, DASS Anxiety Subscale; DASS-Stress, DASS Stress Subscale.

### Planned comparisons

To examine whether there are differences in self-control between OC users and non-users, we collapsed across semesters and conducted a series of independent sample t-tests comparing OC users and non-users on each of the three measures of self-control (see **Figure 1**). To control for multiple comparisons, we utilized a Bonferroni correction, setting alpha at .017 (.05/3). Our findings indicated that individuals using OCs reported significantly higher locomotion (as measured by the Locomotion subscale of the RMS) than those not using OCs, *t*(761.0) = 4.51, *p* <.001, *d* = .27. There were no significant differences between groups on assessment (which we measured using the Assessment subscale of the RMS), *t*(665.4) = 0.54, *p* = .586, *d* = .03 and self-overriding (as measured by the BSCS), *t*(701.4) = 1.01, *p* = .315, *d* = .06.

### Regressions

We also aimed to determine whether oral contraceptive use could predict our measures of self-control over and above symptoms of depression and the semester of data collection. To control for these variables, we conducted a series of hierarchical regressions entering semester and DASS-depression as predictors in the first step and adding oral contraceptive use in the second step. For the sake of brevity, the R^2^ and ΔR^2^ associated with each regression are shown in **Table 2**; full regression tables are available in **Supplementary Materials** (see **Appendix B**).

**Table 2:** Regression model statistics for Study 1.

	R^2^	ΔR^2^	*Model p*	*p for* ΔR^2^
**DV: RMS-Loc** ***Step 1***	.052		<.001	
***Step 2***	.064	.012	<.001	<.001
**DV: RMS-Assess** ***Steps 1***	.061		<.001	
***Step 2***	.061	.000	<.001	.920
**DV: BSCS** ***Step 1***	.147		<.001	
***Step 2***	.147	.000	<.001	.735

Note 1: DV, Dependent variable; BSCS, Brief Self-Control Scale; RMS-Loc, Regulatory Mode Scale – Locomotion Subscale; RMS-Assess, Regulatory Mode Scale – Assessment Subscale.

Note 2: Step 1 included semester of data collection and depression symptoms as predictors. In Step 2, OC use was added to the model.

Note 3: ΔR^2^ may not reflect exact numerical differences in R^2^ values in the table due to rounding.

As can be seen in **Table 2**, entering semester and depression symptoms in Step 1 accounted for a significant amount of overall variance in each outcome variable. More specifically, for locomotion and self-overriding, only depression symptoms (and not semester) accounted for a significant amount of unique variance in Step 1. When predicting assessment, both depression symptoms and semester were unique and significant predictors. In Step 2, OC use explained a significant amount of additional variance (over and above semester and depression) only in locomotion, such that OC use uniquely predicted greater locomotion. OC use did not explain additional variance in assessment or self-overriding (see ΔR^2^ in **Table 2**). After OC use was added in Step 2, semester remained a non-significant predictor for locomotion and self-overriding, while depression symptoms continued to predict unique variance in all outcome measures. Semester continued to only predict significant variance in assessment.

In summary, the findings from Study 1 revealed there were no significant differences between OC users and non-users in terms of self-overriding or self-regulatory assessment (i.e., the evaluation of goals and progress towards them). However, we did find a significant difference between OC users and non-users in self-regulatory locomotion (i.e., movement towards goals), such that those using OCs reported significantly greater locomotion than those not using OCs. These patterns remained even when variance associated with semester of data collection and depression symptoms was statistically partialled out in regression analyses.

## Study 2

In Study 2 we sought to determine whether the results of Study 1 would replicate in an independent sample of undergraduate female.

## Method

### Participants

As in Study 1, all participants were female undergraduates, completed Mass Testing in exchange for partial course credit, and exclusions were made using the criteria outlined in the Data Cleaning section. In Fall 2020, data was collected from 1726 female participants. We excluded 940 participants according to the exclusion criteria outlined in Study 1. This resulted in 786 participants in Fall 2020, with 171 using OCs and 615 naturally cycling. Data was collected from 1573 females in Winter 2021. 1074 participants were excluded (including 443 participants who were already in the Study 1 sample), leaving 499 participants (117 using OCs and 382 naturally cycling). Thus, in total, Study 2 included 1285 participants, with 288 OC users and 997 non-users. Participants were an average of 19 years old. All participated reported their sex as female and included the following gender identities: gender queer/gender non-conforming/gender non-binary (N = 8), man/transman (N = 1), and woman/transwoman (N = 1269). Seven participants did not report their gender identity.

### Pre-screen and mass testing surveys

The Pre-screen and Mass Testing procedure was identical to Study 1 with the exception that data was collected at the beginning of two different semesters: Fall 2020 (September – October) and Winter 2021 (January – February). Participants who were included in the Study 1 sample were excluded if they were also present in the Study 2 sample (see Participants above).

### Data cleaning

The data cleaning procedure was identical to Study 1. As in Study 1, the data cleaning procedure and exclusion criteria were determined a priori. Below, we note the Ns for each exclusion criterion in Study 2. Four hundred and forty-three participants were excluded in Fall 2020 because they were included in Study 1 (in Winter or Spring 2020). We also excluded an additional 189 participants in Winter 2021 because they had been included in the Fall 2020 sample. Forty participants were excluded for using a hormonal contraceptive other than OCs (Fall 2020: N = 22; Winter 2021: N = 18), 158 were excluded because they used a hormonal IUD (Fall 2020: N = 85; Winter 2021: N = 73), 18 due to copper IUD use (Fall 2020: N = 6; Winter 2021: N = 12), or not disclosing whether or not they were using a hormonal contraceptive (N = 213; Fall 2020: N = 112; Winter 2021: N = 101). We also excluded 299 participants currently receiving treatment for depression (Fall 2020: N = 150; OC N = 62, non-OC N = 88; Winter 2021: N = 149; OC N = 54, non-OC N = 95) and 38 for declining to disclose whether or not they were currently receiving treatment for depression (Fall 2020: N = 21; Winter 2021: N = 17). Ninety-three were excluded because they were currently receiving treatment for anxiety (Fall 2020: N = 55; OC N = 27, non-OC N = 28; Winter 2021: N = 38; OC N = 17, non-OC N = 21) and 14 were excluded for not disclosing whether they were currently receiving treatment for anxiety (Fall 2020: N = 9; Winter 2021: N = 5). We excluded 3 participants (Fall 2020: N = 3; Winter 2021: N = 0) using anti-psychotics and 2 who did not disclose anti-psychotic use or non-use (Fall 2020: N = 1; Winter 2021: N = 1) were also excluded. These participants were excluded to avoid mental health issues as a possible confound. Thirty-one participants (Fall 2020: N = 15; Winter 2021: N = 16) were excluded for moving too fast through the survey (we reasoned that participants needed at least one second per item per scale to read the item and respond in good faith). Thus, if they were too quick (i.e., not reading items and likely responding randomly) we excluded them. We also excluded an additional 1 participant (Fall 2020: N = 0; Winter 2021: N = 1) because they completed fewer than 50% of the items on our scales of interest (since we averaged across items to score each scale). Post-hoc, participants who did not report their birth year were removed (N = 26) as well as participants who were older than 45 years of age (N = 3). This ensured our sample consisted of females of reproductive age. Like Study 1, these additional exclusions did not change the interpretation of the results.

## Materials

The materials were identical to those used in Study 1.

## Results and discussion

Analyses were again performed in R (42) using the same packages as Study 1. Anonymized data and analysis scripts are available at https://osf.io/84pc6/. We planned to conduct the same analyses implemented in Study 1 (analyses for Study 2 were pre-registered on OSF, see link above). As before, we compared OC users to non-users on each of the measures of self-control and we employed hierarchical regressions to determine whether OC use could predict our measures of self-control over and above symptoms of depression and the semester of data collection.

### Descriptive statistics

**Figure 2:**
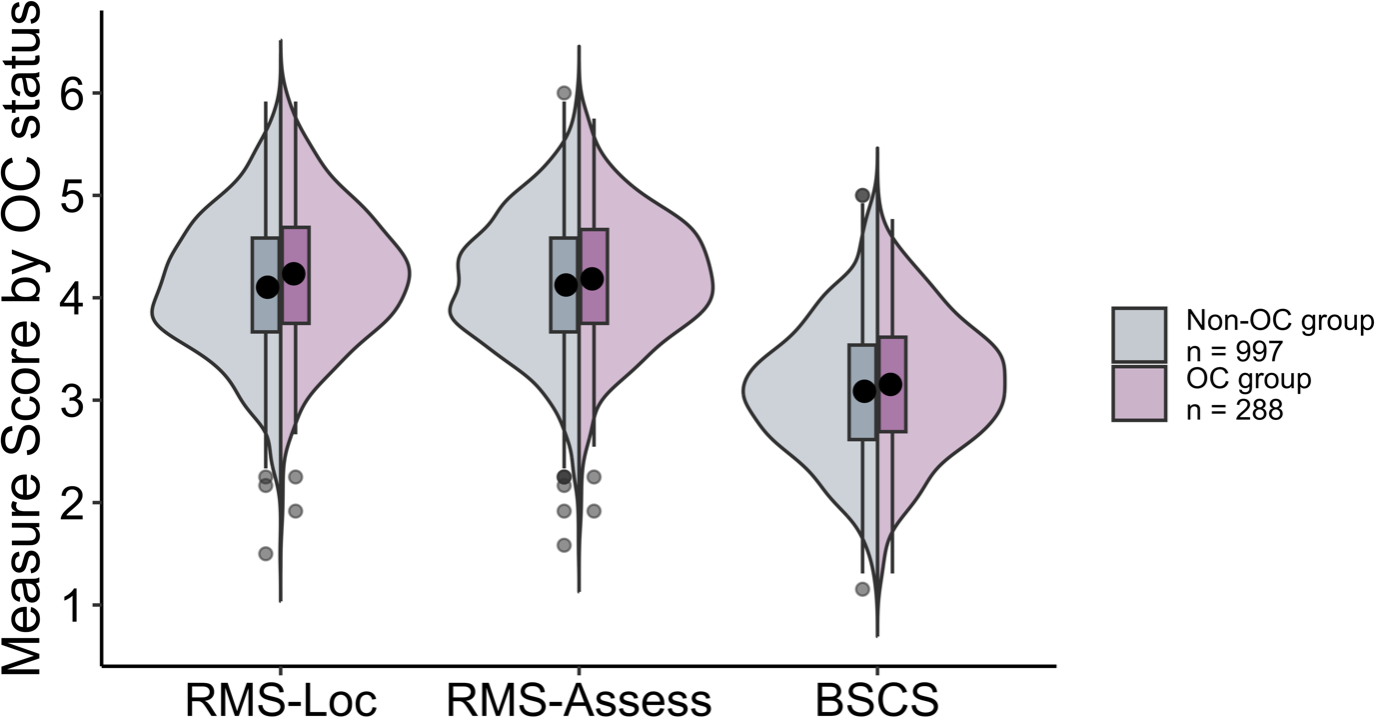
Split violin plot with box and whisker plots (boxplots) for each of the measures (RMS-Locomotion, RMS-Assessment, and BSCS) as a function of OC group (Non-OC vs. OC) in Study 2.

**Table 3** includes the descriptive statistics for both the OC and the non-OC groups. Scales again showed high reliabilities, with Cronbach alphas of .77 or greater (see **Supplementary Materials Table A3**). We also include Pearson correlations between the measures within each group in **Appendix A** of the (**Supplementary Materials Table A4**). Boxplots depicting each of the measures as a function of OC use condition (averaged across semesters) are shown in **Figure 2**.

**Table 3:** Descriptive statistics of measures by semester and group for Study 2.

Semester	Group	Measure	N	Mean	SD	Skew	Kurtosis
Fall 2020	Non-OC group	BSCS	615	3.06	0.66	0.08	-0.17
		RMS-Loc	615	4.09	0.70	-0.10	-0.02
		RMS-Assess	615	4.17	0.67	-0.23	0.16
		DASS-Dep	615	0.93	0.75	0.83	-0.10
		DASS-Anx	615	0.77	0.66	1.02	0.54
		DASS-Stress	615	1.04	0.68	0.38	-0.54
	OC group	BSCS	171	3.18	0.64	-0.09	-0.30
		RMS-Loc	171	4.26	0.65	-0.13	0.08
		RMS-Assess	171	4.19	0.62	-0.54	0.89
		DASS-Dep	171	0.81	0.73	1.00	0.16
		DASS-Anx	171	0.76	0.67	0.92	0.00
		DASS-Stress	171	1.05	0.69	0.56	-0.35
Winter 2021	Non-OC group	BSCS	382	3.13	0.70	0.16	-0.16
		RMS-Loc	382	4.12	0.68	−0.02	−0.39
		RMS-Assess	382	4.05	0.67	-0.03	-0.22
		DASS-Dep	382	0.95	0.76	0.64	-0.50
		DASS-Anx	382	0.74	0.64	0.87	-0.04
		DASS-Stress	382	0.99	0.66	0.36	-0.63
	OC group	BSCS	117	3.12	0.69	-0.14	-0.41
		RMS-Loc	117	4.20	0.74	-0.02	-0.16
		RMS-Assess	117	4.17	0.67	0.14	-0.54
		DASS-Dep	117	0.81	0.74	1.15	0.95
		DASS-Anx	117	0.72	0.70	1.03	0.17
		DASS-Stress	117	1.01	0.66	0.59	-0.28

BSCS, Brief Self-Control Scale; RMS-Loc, Regulatory Mode Scale – Locomotion Subscale; RMS-Assess, Regulatory Mode Scale – Assessment Subscale; DASS-Dep, DASS Depression Subscale; DASS-Anx, DASS Anxiety Subscale; DASS-Stress, DASS Stress Subscale.

### Planned comparisons

Collapsing across semesters with a Bonferroni correction (alpha = .017), we again found that OC users reported significantly greater locomotion on the RMS-Locomotion subscale than non-users, *t*(468.1) = 2.86, *p* = .004, *d* = 0.19. Also as in Study 1, we did not find significant differences between groups on the RMS-Assessment subscale, *t*(482.5) = 1.38, *p* = .167, *d* = 0.09, or the BSCS (i.e., self-overriding), *t*(478.9) = 1.51, *p* = .132, *d* = 0.10.

### Regressions

As in Study 1, we conducted a series of hierarchical regressions predicting the scores on the self-control scales with semester of data collection and DASS-depression as predictors in the first step, and then adding contraceptive use in the second step (see **Table 4**; full regression tables are available in **Supplementary Materials Appendix B**). Together, depression symptoms and the semester of data collection accounted for a significant amount of variance in each measure. Depression predicted unique variance in each measure, while semester only accounted for unique variance in self-regulatory assessment. Similar to Study 1, when considering self-regulatory locomotion as assessed by RMS-Locomotion, we found that adding OC use in Step 2 of the regression accounted for significant additional variance. In this second step, significant unique variance was explained by depression but not semester. Also consistent with Study 1, for self-overriding indexed via the BSCS, we again found that the addition of OC use in Step 2 did not explain additional variance and that in this step depression but not semester of data collection predicted a significant amount of unique variance. Finally, when predicting self-regulatory assessment via the RMS-Assessment subscale we found that the addition of OC use in Step 2 of the regression explained significant additional variance in assessment, whereby OC users reported greater self-regulatory assessment than non-users (see ΔR^2^ in **Table 4**). This outcome differed from that of Study 1, which did not show such an effect. Also, after OC use was added, both depression and semester of data collection continued to explain a significant amount of unique variance in self-regulatory assessment.

**Table 4:** Regression model statistics for Study 2.

	R^2^	ΔR^2^	*Model p*	*p for* ΔR^2^
**DV: RMS-Loc** ***Step 1***	.055		<.001	
***Step 2***	.059	.004	<.001	.020
**DV: RMS-Assess** ***Step 1***	.064		<.001	
***Step 2***	.068	.003	<.001	.039
**DV: BSCS** ***Step 1***	.158		<.001	
***Step 2***	.159	.000	<.001	.594

Note 1: DV, Dependent variable; BSCS, Brief Self-Control Scale; RMS-Loc = Regulatory Mode Scale – Locomotion Subscale, RMS-Assess, Regulatory Mode Scale – Assessment Subscale.

Note 2: Semester of data collection and depression symptoms are entered in Step 1. In Step 2, OC use is added to the model.

Note 3: ΔR^2^ may not reflect exact numerical differences in R^2^ values in table due to rounding.

## General discussion

In the present studies, we investigated whether OC users and non-users differed in their levels of self-control, with a particular focus on self-control defined in terms of the ability to override impulses and the tendency to use two self-regulation strategies in goal attainment, namely, locomotion (the tendency to move towards goals) and assessment (the tendency to evaluate goals and goal attainment options). There were three notable findings. First, in both studies we found that OC users consistently reported significantly greater self-regulatory locomotion than non-users and this relation remained even after partialling out variance associated with semester of data collection and depression symptoms. Second, we found weak evidence of greater self-regulatory assessment for OC users than non-users, which persisted even when semester of data collection and depression were accounted for, but occurred only in Study 2. And third, we consistently found no evidence of differences between groups when it came to self-overriding.

At first glance, these findings may seem discordant with prior work investigating OC use, self-control, and related traits. As mentioned earlier, a randomized control trial by Zethraeus et al. (12) found that participants’ reports of self-control significantly decreased following OC use. Relatedly, Bradshaw et al. (7), showed that hormonal contraceptive use (including OCs) was associated with decreased perseverance [but also see (23)]. In contrast, our study showed OC use was associated with a modest improvement in aspects of self-control (primarily locomotion and perhaps assessment). There are several possible explanations for these apparently conflicting results. For instance, the discordant conclusions may be the result of differences in the way aspects of self-control were operationalized and measured across studies. Our studies included measures of self-control (i.e., self-regulation and self-overriding) that were not measured in prior studies, and as we noted, the relation between OC use and self-control may depend on the precise aspects of self-control measured. There are also substantial differences in sample sizes across studies, and it could be that the results from prior studies with smaller samples may be less stable or robust than results from larger samples, such as those included here. Clearly more work is needed to better understand the reasons for the different conclusions drawn from the available studies.

Returning to our main finding, there are several reasons why OC users may report greater locomotion than non-users. One possible explanation for this correlation is that OC use *causes* greater locomotion. As we mention earlier, there is a dense population of estrogen and progesterone receptors in the PFC (8–11). Given that the PFC has been implicated in self-control (24–26) and that OC users differ from non-users in their PFC connectivity (28, 29) and grey matter volume (43), it is possible these differences in connectivity and morphology might manifest behaviorally as differences in locomotion. On the other hand, it is also possible that the difference in self-regulatory locomotion between OC users and non-users reflects a selection bias whereby women with higher levels of locomotion are more likely to use OCs. Specifically, women higher in self-regulatory locomotion (and perhaps to a lesser extent self-regulatory assessment) may strive to achieve goals that are incompatible with an unplanned pregnancy, which may drive them to be more likely to opt to use OCs. Indeed, the use of OCs could be interpreted as a ‘locomotive action’ used to support goal achievement. Yet another possibility is that the association between OC use and locomotion reflects a survivor effect. According to this account, women with lower levels of self-regulatory locomotion who choose to start taking OCs might be more likely to discontinue use if they experience adverse effects from the OCs. Because these women with lower locomotion end up using OCs for only a short time, they are more likely to be included in the group of non-OC users in a research study than a group of OC users. Given this variety of possible explanations, identifying the precise explanation for the differences in self-regulation between OC and non-OC users should be a primary goal of future studies in this area.

The present findings suggest several additional lines of future research. First, self-regulatory locomotion may be an important third (mediating) variable to consider in studies examining the relation between OC use and various other cognitive functions (e.g., perseverance, sustained attention). Locomotion might serve as a useful mediator because it is closely related to motivation, which is known to be strongly related to cognitive performance (44, 45, Study 3; 46, 47). Second, since locomotion is also positively correlated with conscientiousness, achievement orientation, and decisiveness (31), future work could further examine the relation between OC use and these various traits related to goal achievement. Third, there are dimensions of self-control (e.g., control of emotions) that we did not have the opportunity to consider in the present studies, and future work could focus on exploring whether and how all of the various aspects of self-control are related to OC use. Fourth, while there is a growing interest in the relation between OC use and cognition, there remains a dearth of work on other hormonal (and non-hormonal) contraceptives such as intrauterine devices (IUDs; both hormonal and copper), which have yet to be explored in the context of cognition. Given the growing interest in these devices, assessing their influences on cognition might be another fruitful area for future research.

Finally, there are some limitations to the present findings. First, since participation in our study was optional, it is possible that those who opted to participate had better self-regulation tendencies than those who did not. However, there is no direct evidence of such a selection bias in our data, nor is it clear how such a selection bias would influence any differences between OC users and non-users. Second, because the cohort we studied was a sample of convenience, we unfortunately did not have access to information about the specific brands of OCs participants were using, nor their history of hormonal contraceptive use. Yet if OC use influences aspects of self-regulation via inducing hormonal changes in the body, it would be useful to know if specific hormone formulations and dosing schedules have a greater influence on self-regulation than others. Third, averaging across OC users may be problematic because the effects of some OC formulations might mask or obscure null effects, or even opposite effects, of other OC formulations. Likewise, averaging across menstrual cycle phases in the naturally cycling group may also obscure more nuanced outcomes. Finally, we did not collect participants’ histories of OC use, pregnancy, and gynecological disorders. These might be important because they might influence hormonal levels in the body and/or brain physiology, which may in turn have knock-on effects on the relation between OC use and self-control. Given these limitations, future explorations of the roles of OC formulation and reproductive history might shed light on the mechanism underlying the relation between OC use and self-control. As these various avenues of future research are pursued, we will undoubtably learn more about the interesting and nuanced relation between hormonal contraceptive use and cognition.

## Data availability statement

The datasets presented in this study can be found in online repositories. The names of the repository/repositories and accession number(s) can be found below: https://osf.io/84pc6/.

## Ethics statement

The studies involving humans were approved by a University of Waterloo Research Ethics Committee (ORE #44980). The studies were conducted in accordance with the local legislation and institutional requirements. The participants provided their written informed consent to participate in this study.

## Author contributions

AS: Conceptualization, Data curation, Formal Analysis, Investigation, Methodology, Writing – original draft, Writing – review & editing. DS: Conceptualization, Funding acquisition, Investigation, Methodology, Supervision, Writing – review & editing.

## Footnotes

1 Three items assessed self-control: “I was emotionally stable and sure of myself during the past month” (rated from 0 = none of the time to 5 = all of the time), “Have you been in firm control of your behavior, thoughts, emotions or feelings during the past month?” (rated from 0 = yes, definitely so to 5 = no, and I am very disturbed), and “Have you had any reason to wonder if you were losing your mind, or losing control over the way you act, talk, think, feel or of your memory during the past month?” (rated from 0 = not at all to 5 = yes, very much so and I am very concerned).

2 The IUD item did not differentiate between hormonal and copper in the Winter and Spring 2020 semesters.

3 We also compared depression, anxiety, and stress symptoms across OC and non-OC groups. However, since this was not a research question of interest in the current paper, these analyses are included in the **Supplementary Materials** (**Appendix C**).
