# Supplementary material for: On the relation between oral contraceptive use and self-control
Source: Front Endocrinol (Lausanne). 2024 Apr 2;15:1335384. doi: 10.3389/fendo.2024.1335384 (PMC11018928; doi:10.3389/fendo.2024.1335384)
Supplement: Supplementary file 1 [file DataSheet_1.docx]

**Supplementary Materials**

**Appendix A**

**Study 1**

*Table A1 Cronbach alphas*

| *Measure* | *OC group*  *(N = 399)* | *Non-OC group*  *(N = 964)* |
| --- | --- | --- |
| 1. BSCS | .87 | .84 |
| 2. RMS-Assess | .82 | .76 |
| 3. RMS-Loc | .83 | .83 |
| 4.DASS-Depression | .92 | .91 |
| 5. DASS-Anxiety | .81 | .82 |
| 6. DASS-Stress | .87 | .85 |

*Note*: BSCS = Brief Self-Control Scale; RMS-Assess = Regulatory Mode Scale – Assessment subscale; RMS-Loc = Regulatory Mode Scale – Locomotion subscale.

*Table A2 Pearson correlations of the OC group (above the diagonal; N=399) and non-OC group (below the diagonal; N = 964)*

| *Measure* | *1* | *2* | *3* | *4* | *5* | *6* |
| --- | --- | --- | --- | --- | --- | --- |
| 1. RMS-Loc | -- | .10 * | .45 ** | -.19 ** | -.15 * | -.08 |
| 2. RMS-Assess | .16 ** | -- | -.27 ** | .28 ** | .26 ** | .37 ** |
| 3. BSCS | .40 ** | -.19 ** | -- | -.41 ** | -.32 ** | -.38 ** |
| 4.DASS-Depression | -.23 ** | .22 ** | -.37 ** | -- | .68 ** | .77 ** |
| 5. DASS-Anxiety | -.07 * | .23 ** | -.27 ** | .68 ** | -- | .73 ** |
| 6. DASS-Stress | -.03 | .28 ** | -.34 ** | .74 ** | .76 ** | -- |

*Note*: BSCS = Brief Self-Control Scale; RMS-Assess = Regulatory Mode Scale – Assessment subscale; RMS-Loc = Regulatory Mode Scale – Locomotion subscale.

* p = .05, ** p < .001

**Study 2**

*Table A3 Cronbach alphas*

| *Measure* | *OC group*  *(N = 288)* | *Non-OC group*  *(N = 997)* |
| --- | --- | --- |
| 1. BSCS | .83 | .84 |
| 2. RMS-Assess | .77 | .78 |
| 3. RMS-Loc | .83 | .82 |
| 4.DASS-Depression | .91 | .90 |
| 5. DASS-Anxiety | .84 | .81 |
| 6. DASS-Stress | .84 | .84 |

*Note*: BSCS = Brief Self-Control Scale; RMS-Assess = Regulatory Mode Scale – Assessment subscale; RMS-Loc = Regulatory Mode Scale – Locomotion subscale.

*Table A4 Pearson correlations of the OC group (above the diagonal; N=288) and non-OC group (below the diagonal; N = 997)*

| *Measure* | *1* | *2* | *3* | *4* | *5* | *6* |
| --- | --- | --- | --- | --- | --- | --- |
| 1. RMS-Loc | -- | .10 | .46 ** | -.29 ** | -.14 * | -.14 * |
| 2. RMS-Assess | .16 | -- | -.18 ** | .20 ** | .25 ** | .29 ** |
| 3. BSCS | .44 ** | -.20 ** | -- | -.38 ** | -.30 ** | -.37 ** |
| 4.DASS-Depression | -.21 ** | .26 ** | -.40 ** | -- | .64 ** | .75 ** |
| 5. DASS-Anxiety | -.06 * | .19 ** | -.27 ** | .63 ** | -- | .78 ** |
| 6. DASS-Stress | -.01 * | .29 ** | -.27 ** | .69 ** | .75 ** | -- |

*Note*: BSCS = Brief Self-Control Scale; RMS-Assess = Regulatory Mode Scale – Assessment subscale; RMS-Loc = Regulatory Mode Scale – Locomotion subscale.

* p = .05, ** p < .001

**Appendix B**

**Study 1**

**Regressions**

As noted in the manuscript, full regression model statistics for Study 1 are presented below.

Table B1 Regression predicting locomotion (*N* = 1363) by the term, DASS-Depression, and oral contraceptive use

| Predictors | R^2^ | ΔR^2^ | *F* | SE | b | *t* | *p* |
| --- | --- | --- | --- | --- | --- | --- | --- |
| *Step 1* | **.052** |  | **37.33** | **0.67** |  |  | **< .001** |
| Constant |  |  |  |  | 4.02 | 195.49 | < .001 |
| Semester - Spring |  |  |  |  | 0.06 | 1.47 | .143 |
| DASS-Depression |  |  |  |  | -0.16 | 8.51 | < .001 |
| *Step 2* | **.064** | **.012** | **30.94** | **0.67** |  |  | **< .001** |
| Constant |  |  |  |  | 3.97 | 168.42 | < .001 |
| Semester - Spring |  |  |  |  | 0.07 | 1.52 | .129 |
| DASS-Depression |  |  |  |  | -0.15 | 8.34 | < .001 |
| Birth control – OC use |  |  |  |  | 0.17 | 4.15 | < .001 |

*Note 1.* The statistics for the overall model can be found in bold, to the right of Step 1 and Step 2 in the table above. Statistics for the model change can be found in-text in the results section of the manuscript.

Note 2. ΔR^2^ may not reflect exact numerical differences in R^2^ values in table due to rounding.

Table B2 Regression predicting assessment (*N* = 1363) by the term, DASS-Depression, and oral contraceptive use

| Predictors | R^2^ | ΔR^2^ | *F* | SE | b | *t* | *p* |
| --- | --- | --- | --- | --- | --- | --- | --- |
| *Step 1* | **.061** |  | **44.44** | **0.65** |  |  | **< .001** |
| Constant |  |  |  |  | 4.00 | 201.29 | < .001 |
| Term - Spring |  |  |  |  | 0.08 | 1.97 | .048 |
| DASS-Depression |  |  |  |  | 0.16 | 9.22 | < .001 |
| *Step 2* | **.061** | **.000** | **29.61** | **0.65** |  |  | **< .001** |
| Constant |  |  |  |  | 4.00 | 174.50 | < .001 |
| Term - Spring |  |  |  |  | 0.08 | 1.98 | .048 |
| DASS-Depression |  |  |  |  | 0.16 | 9.20 | < .001 |
| Birth control – OC use |  |  |  |  | -0.00 | 0.10 | .920 |

*Note 1.* The statistics for the overall model can be found in bold, to the right of Step 1 and Step 2 in the table above. Statistics for the model change can be found in-text in the results section of the manuscript.

Note 2. ΔR^2^ may not reflect exact numerical differences in R^2^ values in table due to rounding.

Table B3 Regression predicting self-control (*N* = 1363) by the term, DASS-Depression, and oral contraceptive use

| Predictors | R^2^ | ΔR^2^ | *F* | SE | b | *t* | *p* |
| --- | --- | --- | --- | --- | --- | --- | --- |
| *Step 1* | **.147** |  | **116.80** | **0.61** |  |  | **< .001** |
| Constant |  |  |  |  | 3.11 | 166.47 | < .001 |
| Term - Spring |  |  |  |  | 0.01 | 0.36 | .718 |
| DASS-Depression |  |  |  |  | -0.25 | 15.28 | < .001 |
| *Step 2* | **.147** | **.000** | **77.83** | **0.61** |  |  | **< .001** |
| Constant |  |  |  |  | 3.11 | 144.11 | < .001 |
| Term - Spring |  |  |  |  | 0.01 | 0.37 | .715 |
| DASS-Depression |  |  |  |  | -0.25 | 15.23 | < .001 |
| Birth control – OC use |  |  |  |  | 0.01 | 0.34 | .735 |

*Note 1.* The statistics for the overall model can be found in bold, to the right of Step 1 and Step 2 in the table above. Statistics for the model change can be found in-text in the results section of the manuscript.

Note 2. ΔR^2^ may not reflect exact numerical differences in R^2^ values in table due to rounding.

**Study 2**

**Regressions**

Like Study 1 above, we present full regression model statistics for Study 2 below.

Table B5 Regression predicting locomotion (*N* = 1285) by the term, DASS-Depression, and oral contraceptive use

| Predictors | R^2^ | ΔR^2^ | *F* | SE | b | *t* | *p* |
| --- | --- | --- | --- | --- | --- | --- | --- |
| *Step 1* | **.055** |  | **37.34** | **0.67** |  |  | **< .001** |
| Constant |  |  |  |  | 4.12 | 171.69 | < .001 |
| Term - Winter 2021 |  |  |  |  | 0.02 | 0.43 | .669 |
| DASS-Depression |  |  |  |  | -0.16 | 8.63 | < .001 |
| *Step 2* | **.059** | **.004** | **26.79** | **0.67** |  |  | **< .001** |
| Constant |  |  |  |  | 4.10 | 158.33 | < .001 |
| Term – Winter 2021 |  |  |  |  | 0.01 | 0.38 | .704 |
| DASS-Depression |  |  |  |  | -0.16 | 8.47 | < .001 |
| Birth control – OC use |  |  |  |  | 0.11 | 2.33 | .020 |

*Note 1.* The statistics for the overall model can be found in bold, to the right of Step 1 and Step 2 in the table above. Statistics for the model change can be found in-text in the results section of the manuscript.

Note 2. ΔR^2^ may not reflect exact numerical differences in R^2^ values in table due to rounding.

Table B6 Regression predicting assessment (*N* = 1285) by the term, DASS-Depression, and oral contraceptive use

| Predictors | R^2^ | ΔR^2^ | *F* | SE | b | *t* | *p* |
| --- | --- | --- | --- | --- | --- | --- | --- |
| *Step 1* | **.064** |  | **44.17** | **0.64** |  |  | **< .001** |
| Constant |  |  |  |  | 4.17 | 181.97 | < .001 |
| Term - Winter 2021 |  |  |  |  | -0.09 | 2.57 | .010 |
| DASS-Depression |  |  |  |  | 0.16 | 9.07 | < .001 |
| *Step 2* | **.068** | **.003** | **30.95** | **0.64** |  |  | **< .001** |
| Constant |  |  |  |  | 4.15 | 167.88 | < .001 |
| Term - Winter 2021 |  |  |  |  | -0.09 | 2.61 | .009 |
| DASS-Depression |  |  |  |  | 0.17 | 9.20 | < .001 |
| Birth control – OC use |  |  |  |  | 0.09 | 2.07 | .039 |

*Note 1.* The statistics for the overall model can be found in bold, to the right of Step 1 and Step 2 in the table above. Statistics for the model change can be found in-text in the results section of the manuscript.

Note 2. ΔR^2^ may not reflect exact numerical differences in R^2^ values in table due to rounding.

Table B7 Regression predicting self-control (*N* = 1285) by the term, DASS-Depression, and oral contraceptive use

| Predictors | R^2^ | ΔR^2^ | *F* | SE | b | *t* | *p* |
| --- | --- | --- | --- | --- | --- | --- | --- |
| *Step 1* | **.158** |  | **120.70** | **0.62** |  |  | **< .001** |
| Constant |  |  |  |  | 3.09 | 139.55 | < .001 |
| Term - Winter 2021 |  |  |  |  | 0.04 | 1.23 | .221 |
| DASS-Depression |  |  |  |  | -0.27 | 15.50 | < .001 |
| *Step 2* | **.159** | **.000** | **80.50** | **0.62** |  |  | **< .001** |
| Constant |  |  |  |  | 3.08 | 128.94 | < .001 |
| Term - Winter 2021 |  |  |  |  | 0.04 | 1.21 | .225 |
| DASS-Depression |  |  |  |  | -0.27 | 15.42 | < .001 |
| Birth control – OC use |  |  |  |  | 0.02 | 0.53 | .594 |

*Note 1.* The statistics for the overall model can be found in bold, to the right of Step 1 and Step 2 in the table above. Statistics for the model change can be found in-text in the results section of the manuscript.

Note 2. ΔR^2^ may not reflect exact numerical differences in R^2^ values in table due to rounding.

**Appendix C**

**Study 1**

**DASS Scores**

To determine if there were differences between groups on symptoms of depression, anxiety, and stress, we conducted a series of independent samples t-tests. We found no significant differences between groups on symptoms of depression, *t*(745.7) = 1.89, *p* = .059, *d* = .11, however those using OCs reported nominally fewer depression symptoms than non-OC users. There were also no differences between groups on either symptoms of anxiety, *t*(758.0) = 1.13, *p* = .257, *d* = .07, or symptoms of stress, *t*(713.8) = 0.90, *p* = .369, *d* = .05.

**Study 2**

**DASS Scores**

To investigate whether there were differences in symptoms of depression, anxiety, and stress between those using OCs and those not, we conducted a series of independent samples t-tests. Our findings indicated that OC users reported significantly fewer depression symptoms than non-OC users, *t*(479.2) = 2.50, *p* = .013, *d* = .16. We did not find differences in symptoms of anxiety, *t*(451.7) = 0.44, *p* = .662, *d* = .03, nor symptoms of stress, *t*(460.3) = 0.23, *p* = .819, *d* = .02.
